# A scoping review to inform an auditing framework evaluating healthcare environments for inclusion of people with intellectual disability and/or autism

**DOI:** 10.1177/17446295231174282

**Published:** 2023-05-22

**Authors:** Michelle Kersten, Nathan John Wilson, Amy Pracilio, Virginia Howie, Julian Trollor, Thomas Buckley, Julia Morphet, Julianne Bryce, Ken Griffin, Andrew Cashin

**Affiliations:** Faculty of Health and Human Sciences, 4571 Southern Cross University, Lismore, NSW, Australia; School of Nursing and Midwifery, 6489 Western Sydney University, Penrith South, NSW, Australia; Faculty of Health and Human Sciences, 4571 Southern Cross University, Lismore, NSW, Australia; Department of Developmental Disability Neuropsychiatry, UNSW Medicine and Health, 7800 UNSW Sydney, Sydney, NSW, Australia; Susan Wakil Building, Faculty of Medicine and Health, University of Sydney, Sydney, NSW, 2006, Australia; Nursing & Midwifery, Monash University, Peninsula Campus, Frankston, VIC, 3199, Australia; Australian Nursing and Midwifery Federation, Federal Office, Melbourne, VIC, 3000, Australia; Australian Primary Health Care Nurses Association, Melbourne, VIC, 3000, Australia; Faculty of Health and Human Sciences, 4571 Southern Cross University, Lismore, NSW, Australia

**Keywords:** healthcare services, evaluation, intellectual disability, autism, equity, reasonable adjustments

## Abstract

People with intellectual disability and/or autism are likely to be in hospital more often, for longer, and have poorer health outcomes. Few audit tools exist to identify their barriers in mainstream healthcare environments. This study aimed to identify evidence of audit characteristics of healthcare contexts specifically for people with intellectual disability and/or autism, for conceptual development of an auditing framework. A scoping review of evaluations of healthcare environments was completed in January 2023. Findings were presented using the PAGER framework. Of the sixteen studies identified, most originated in the UK, nine focused on intellectual disability, four on autism, and three were concerned with mixed diagnosis. Six domains for auditing healthcare environments were identified: care imperatives, communication to individuals, understanding communication from individuals, providing supportive environments of care, supporting positive behaviour, and actions to make things go well. Further research is recommended to refine an audit framework.

## Introduction

Interacting with healthcare environments can be challenging for people with intellectual disability and /or autism, with the potential to adversely affect health outcomes (Doherty et al., 2020). They are likely to be in hospital more often, for longer periods, and have poorer health outcomes than people without disability (Trollor et al., 2017). Although there is a tendency for research to focus on healthcare users with either intellectual disability or autism this paper jointly considers both intellectual disability or autism, or dual diagnosis of both. There is heterogeneity of the criterion for dual diagnosis internationally, but we refer to people who have received a formal diagnosis of both. All three groups share similar barriers in a range of mainstream acute, primary, and ambulatory healthcare environments – hereafter referred to as healthcare environments - due to difficulty understanding information and communication issues, though these present in different ways. For example, people with autism often report difficulties with anxiety, information processing, and communicating in healthcare environments (Raymaker et al., 2017). Likewise, people with intellectual disability frequently have difficulty communicating and understanding information in health contexts (Mastebroek et al., 2014), and may exhibit behavioural characteristics similar to autism (Thurm et al., 2019).

If healthcare is to be truly inclusive, and health outcomes improved, it is crucial to recognise how healthcare environments interact with person factors to influence equitable access to healthcare for people with intellectual disability and autism. Adjustments to healthcare environments for people with mobility difficulties, low vision, or hearing impairment are addressed by mandated universal access standards in many countries. An example of these are the Australian Standards for the Design of Access and Mobility (Standards Australia, 2009), applied to new public buildings. However, the environmental barriers within healthcare environments requiring consideration for healthcare users with intellectual disability and/or autism go beyond physical environments (Doherty et al., 2020; Tuffrey-Wijne et al., 2014). The International Classification of Functioning (ICF) (World Health Organization, 2001) provides a conceptualisation of the environment into five broad factors: i) environments arising from products and technology, ii) the natural environment and human made changes to environment, iii) support and relationships, iv) attitudes, and finally v) services, systems, and policies (Schneidert et al., 2003). This conceptualisation helps unpack and frame barriers present in healthcare for people with intellectual disability, people with autism, or dual diagnosis.

### How do healthcare environments impact health for people with intellectual disability and/or autism?

People with intellectual disability and/or autism are impacted by the health care environment partly due to sensory, communication, and cognitive difficulties (Calleja et al., 2020; Doherty et al., 2020). Recently, barriers to healthcare services experienced by people with autism (Mason et al., 2019; Walsh et al., 2020; Calleja et al., 2020) or with intellectual disability (Trollor et al., 2017; Calleja et al., 2020), have been examined, in recognition of their disparately poorer health outcomes (Cashin et al., 2018). Understanding the dynamic relationships between healthcare environments and the unique needs of people with intellectual disability and/or autism offers opportunities for providing reasonable adjustments that decrease healthcare barriers and improve health outcomes. Reasonable adjustments include both changes needed to care for an individual, or anticipatory changes for a population of people with specific disabilities (Marsden and Giles, 2017). These could involve changes to practices, policies or procedures for either groups or individuals, that support equitable access to healthcare (Heslop et al., 2019).

In consideration of the ICF category of environments arising from *products and technology*, this review has been unable to identify studies detailing specific technological barriers for people with intellectual disability and/or autism. However, technological and product barriers could arguably include being able to bring assistive technology used at home, such as communication or seating/positioning equipment, or the accessibility of healthcare equipment such as hospital beds or call bells. Of note is the potential benefits of technology, for example, telehealth services have delivered health access improvements for people with intellectual disability (Krysta et al., 2021) and autism (Knutsen et al., 2016). The increased use of telehealth services by people with intellectual disability and autism during the COVID-19 pandemic has been explored in several international studies which note how technology ensured the provision, continuation, and accessibility of health services (Friedman, 2022; Pelligrino and Di Gennaro Reed, 2021; Rawlings et al., 2021).

The *natural environment and human made* aspects of healthcare environments can also pose barriers to equitable access to healthcare. Barriers for people with autism can relate to sensory stimuli, such as difficulty tolerating busy waiting room environments or being touched for physical examination (Mason et al., 2019); people and machine noise, bright or overstimulating lighting and cluttered environments (Walsh et al., 2020); or odour (Muskat et al., 2015). The sensory sensitivities in waiting rooms and the uncertainty of waiting for healthcare has been identified as particularly challenging by both adults (Raymaker et al., 2017) and children (Wood et al., 2019) with autism. Reasonable adjustments to healthcare environments can reduce sensory overload for people with autism by offering low stimulation environments, for example, removing fluorescent lighting, provision of sound-proof rooms, soothing music, and halting loudspeaker announcements (Howie et al., 2021). Many people with intellectual disability have comorbid hearing or vision impairment as well as physical impairment, making it an added challenge to navigate healthcare environments from multiple dimensions, such as wayfinding (Howie et al., 2021). Additionally, people with intellectual disability who require very high levels of care may need wheelchair access and adult change facilities (Martin, 2013).

The *support and relationships* fostered in healthcare environments, are clearly documented barriers to equitable healthcare access. For people with autism these can include difficulty in social interaction with many unfamiliar people (Walsh et al., 2020) and communication with healthcare professionals (Mason et al., 2019). Similarly, for people with intellectual disability, healthcare professionals identify communicating with people with intellectual disability and understanding the caregiver role within healthcare as challenging (Lewis et al., 2017). Collaborating with caregivers was found to be important for communicating accurate health information to families of children with autism (Walsh et al., 2020), for services to identify reasonable adjustment needs and for caregivers to prepare children for a successful healthcare experience (Muskat et al., 2015). Collaborating with caregivers of people with intellectual disability can assist with navigating access to care, plan reasonable adjustments (Ali et al., 2013) and assist with communication challenges (Hemsley et al., 2011).

Significantly, the *attitudes* towards people with intellectual disability and/or autism are well documented barriers to equitable healthcare. Two systematic reviews identified that healthcare providers without knowledge of the needs of people with autism can be dismissive of caregiver concerns and experiences, be unwilling to make reasonable adjustments (Walsh et al., 2020), or make inaccurate assumptions about the person with autism (Mason et al., 2019). Likewise, people with intellectual disability can also experience discrimination, stigmatisation and poor attitudes from healthcare workers, resulting in poor health outcomes (Pelleboer-Gunnink et al., 2017).

Finally, and arguably the most important environmental factors are those arising from *the services, systems and policies of healthcare environments.* For people with autism, barriers include long waiting times for consultation, and the inherent uncertainty of busy healthcare environments (Walsh et al., 2020). For people with intellectual disability, system issues can include having difficulty with the complexity of navigating access to services, or not being able to find appropriate services because they fall outside of service eligibility criteria (Ali et al., 2013).

### Auditing healthcare environments

Healthcare environments frequently use an auditing process to monitor and improve healthcare delivery (Foy et al., 2020). Despite multiple audit tools designed to evaluate the general quality of healthcare environments (Andrade et al., 2012), a systematic review of instruments measuring healthcare environmental quality identified that many existing audit tools focus on aged care environments (Elf et al., 2017). It is important to determine how the range of healthcare environments are currently supporting the inclusion of people with intellectual disability and/or autism, to monitor and stimulate progress. Walsh et al. (2020) called for development of an audit tool of healthcare environments after completing a systematic review of the physical barriers experienced by adults and children with autism, after reviewing 31 studies, and triangulating data with three stakeholder groups. They proposed a taxonomy of four main healthcare barriers: challenges associated with autism characteristics, healthcare provider-based issues, healthcare system issues, and social environment and attitudes.

Currently there are no comprehensive audit tools published to evaluate equitable access to healthcare environments for people with intellectual disability and/or autism. This scoping review aimed to systematically identify and map the breadth of evidence available concerning the characteristics of existing audits of healthcare contexts for people with intellectual disability and/or autism, particularly relating to environmental factors. Scoping reviews are a tool for gathering evidence about a research question, however, the needs of the audience must be considered when reporting findings (Bradbury-Jones et al., 2021). As applied researchers, we aimed to fill the gap identified via a scoping review of existing audit processes, to propose holistic audit domains, that can be tested in a future study. The scoping review therefore was used to inform the conceptual development of an auditing framework, focused specifically on evaluating the environments of healthcare, with the broader ambition of improving healthcare access, equity, and improving health outcomes, for people with intellectual disability and/or autism.

## Methods

The PRISMA-ScR (Preferred Reporting Items for Systematic Reviews) extension for scoping reviews provided a methodology for systematically searching literature, charting data, and synthesis of healthcare environment factors audited (Tricco et al., 2018). The findings are presented using the PAGER (Patterns, Advances, Gaps, Evidence for practice and Research recommendations) framework which extends PRISMA-ScR methodology to structure reporting of findings (Bradbury-Jones et al., 2021). Therefore the scoping review findings and discussion are organised into a description of the patterns in the data, advances in knowledge and gaps in knowledge. The proposed conceptual audit domains recommended for further research development are then presented, underpinned by the evidence identified via the scoping review.

### Scoping review search strategy

**Figure 1.**
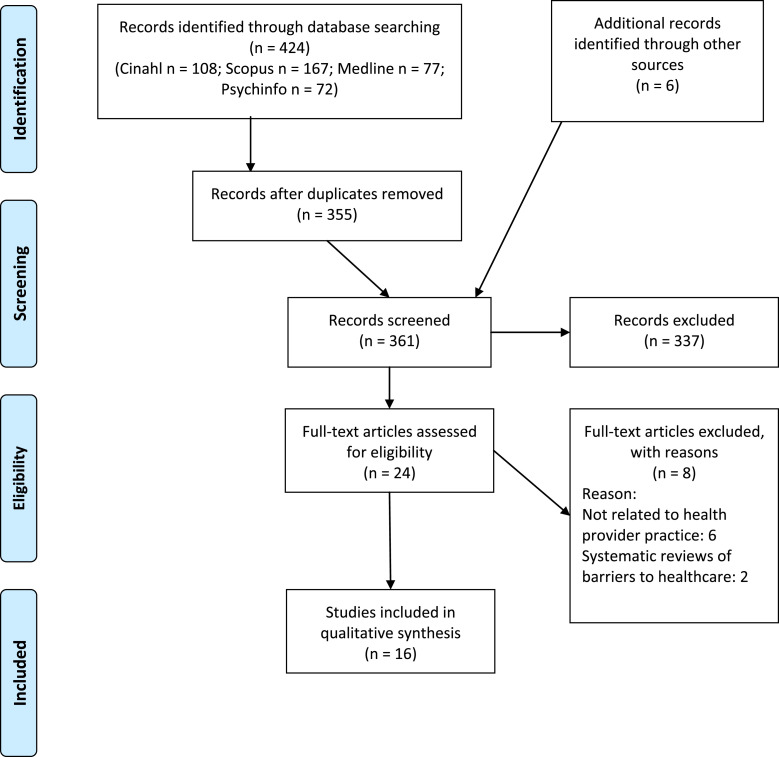
Prisma flow 2009 diagram of the study selection process. Adapted from Moher, D., Liberati, A., Tetzlaff, J., Altman, D., & The PRISMA Group. (2009). Preferred Reporting Items for Systematic Reviews and Meta-Analyses: The PRISMA Statement. Plos Medicine, 6(7), e1000097.

The scoping review research question was “what are the characteristics of formal audits or evaluations of healthcare contexts or environments, reported in published studies, for populations with intellectual disability and/or autism”. Scoping reviews are strengthened by a team based approach to provide content expertise (Westphaln et al., 2021), therefore search terms and article inclusion criteria were developed by the expert project team based on the patient, intervention, comparison, outcome (PICO) model, available in Table 1 (Cooke et al., 2012). The literature was systematically searched during January 2023 in four relevant databases: CINAHL, Ovid MEDLINE, and Psych INFO, and SCOPUS for peer reviewed studies in English published from 2010 to January 2023. Search terms were refined to ensure the search was comprehensive and tailored to the requirements of each database, available in Table 2. Search terms included *intellectual disability, developmental disability, autistic disorder, Asperger syndrome*, *learning disability* or *mental handicap*, combined in various forms with record review, nursing audit, audit, practice audit, context audit or environ* audit. A summary of the PRISMA search is available in Figure 1.

**Table 1.** Study search terms and inclusion criteria.

Population	Intellectual disability &/ or autism spectrum disorder
Phenomena of interest	Evaluation of nursing & relevant healthcare services
Context	Healthcare environments, including specialist services, hospitals & primary care settings
Outcome	Evaluation of the delivery or accessibility of healthcare within health care environments, excluding narrow focus on disease/illness or the pharmaceutical management disease/illness
Study Design	All relevant studies were considered, including quantitative, qualitative, or mixed methods designs
Journal type	All studies published in peer-reviewed journals
Language	English only
Date range	2010-January 2023

**Table 2.** Database search terms.

Database	Search term
Ovid MEDLINE	exp Autistic Disorder/exp Asperger Syndrome/ or Autism Spectrum Disorder/Intellectual Disability/exp Learning Disabilities/(intellectual disabil* or learning disabil* or autis*).mp.
	exp Medical Audit/ or exp Clinical Audit/ or Checklist/ or audit.mp.
	exp Health Services Accessibility/ or Professional Practice/ or Practice Guidelines as Topic/ or exp Nurses/ or exp Primary Health Care/ or exp “Delivery of Health Care”/ or exp Patient Care Team/
CINAHL	(MH “Record Review”) OR (MH “Nursing Audit”) OR (MH “Audit”) OR “audit” OR “practice audit” or “context audit” OR “environ* audit”
	(MH “Health Services for Persons with Disabilities”) OR (MH “Health Services Accessibility”) OR (MH “Professional Practice”) OR (MH “Nursing Practice”) OR (MH “Nursing Staff, Hospital”) OR (MH “Staff Nurses”) OR (MH “Primary Health Care”) OR (MH “Multidisciplinary Care Team”) OR (MH “Health Care Delivery, Integrated”)
	(MH “Intellectual Disability”) OR (MH “Developmental Disabilities”) OR “learning disabil*” OR “intellectual disabil*” OR (MH “Autistic Disorder”) OR (MH “Asperger Syndrome”) OR autis*
Psych INFO	Autism Spectrum Disorders/Intellectual Development Disorder/ or Learning Disabilities/ or Developmental Disabilities/(intellectual disabil* or learning disabil* or autis*).mp.
	exp Clinical Audits/ or exp “Checklist (Testing)”/ or audit.mp. exp Health Care
	Services/ or exp Health Care Delivery/ or exp Health Care Access/ exp Primary Health Care/ or Nurses/
Scopus	“intellectual disabil*” or “learning disabil*” or autis* or (asperger* and audit or “record review”) and (“health service*” or “primary health care” or “health access” or “health care access”

Inclusion and exclusion criteria for selection of sources were developed by the project team. Studies were included if they: (1) evaluated all healthcare contexts, including mainstream acute, primary and ambulatory healthcare contexts as well as any specialised disability-specific healthcare context, (2) in English, (3) published in a peer reviewed journal, and (4) for any age. Studies were excluded if they: (1) did not evaluate the healthcare environment, (2) were not specific to intellectual disability and/or autism and (3) if they narrowly focussed on a disease/illness or the pharmaceutical management of a disease/illness.

The initial screening was completed by the first author. Following this, 24 full text records were independently assessed for inclusion/exclusion, by two authors. Any disagreements were discussed until consensus was achieved. This resulted in 16 studies meeting the criteria for analysis.

### Data charting process

Data from the final 16 studies were extracted by the first author to create a descriptive summary of the results relevant to the scoping review's research question and study aim, following piloting of the data extraction form with the second and tenth authors. Data extracted included year of study, country, study aims, number of participants, context or setting, methods, and relevant findings of the study (see supplementary Table), and audit items and characteristics (see Table 3), and was reviewed by the second and tenth authors.

**Table 3.** Factors identified in the audit of healthcare settings.

Key audit factors Author/year/country	Context/ Setting	A. Communication	B. Working together with families/caregivers	C. Supporting decision making	D. Adjusting environments of care	E. Systematic identification to trigger needs assessment	F. Supporting positive behaviour	G. Staff knowledge	H. Having resources for care	I. Management of physical/ medical care	J. Care management
Chilvers et al. (2013), UK	Outpatient specialist service for children with developmental delay/mental health	✓	✓	✓					✓		✓
Cocquyt (2018), UK	Regional community adult service for ID	✓			✓					✓	✓
da Costa et al. (2011), UK	Specialist community service for ID	✓								✓	✓
Davies et al. (2019), UK	Specialist mental health unit for ID		✓			✓	✓	✓	✓		
Esan and Marker (2010), UK	Specialist outpatient epilepsy unit for ID	✓			✓					✓	✓
Harrison and Willis (2015), UK	General antenatal services and ID				✓	✓					✓
Kennedy et al. (2016), UK	Children’s hospital and autism		✓			✓		✓			✓
Marsden & Giles (2017), UK	Acute general hospital and ID	✓	✓	✓							✓
Penney and Blair (2020), UK	Specialist community service for ID				✓		✓	✓	✓	✓	✓
Pratt et al. (2012), UK	Children’s hospital and ID/autism/ challenging behaviour	✓			✓	✓	✓				✓
Raymaker et al. (2017), US	General healthcare and autism	✓	✓	✓	✓		✓	✓			✓
Saqr et al. (2018), US	Specialist primary healthcare for adults with autism	✓	✓		✓		✓			✓	✓
Shardlow & Thalayasingam (2010), UK	Inpatient specialist long stay residential service for ID	✓			✓			✓	✓	✓	✓
Sheehan et al. (2016), UK	General hospital/ mental health and ID	✓	✓	✓						✓	✓
Simpson (2020), UK	General healthcare and autism	✓			✓			✓			
Tromans et al. (2019)	End of life care in community/ inpatient mental health and ID		✓	✓				✓		✓	

### Data analysis

Data regarding audit characteristics were analysed thematically by the first author, and organised to form broad groupings of factors represented by the audit items. Analysis was reviewed by the second and tenth author, and discussed by the project team. From these factors, audit domains were developed iteratively through regular project team discussion. From these emerged the conceptual framework of audit domains for auditing mainstream healthcare environments for people with intellectual disability and/or autism, proposed in the evidence for practice and recommendations for research section.

## Findings

### Patterns identified in the reviewed studies

Of the 16 studies selected for review, described in detail in the supplementary table, most originated in the UK (n=14), with less focus from the US (n=2). The US studies focused on autism in general/mainstream healthcare. Ten audits of healthcare environments in the UK referred to UK specific guidelines/standards/toolkits such as the National Institute for Health and Care Excellence (NICE) standards (n=4), Royal College of Psychiatrists standards (n=2), The Positive Behaviour Support Academy (n=1), Safe, Clean and Personal Every time Guidelines (SCAPE) n=1, or other UK specialist services guidelines, such as end of life care or maternity care (n=2). All studies focusing on intellectual disability originated from the UK.

Of the 16 studies selected for review, nine focused on intellectual disability, four focused on autism, and three included dual diagnoses of intellectual disability, autism, or other developmental disability. The 16 studies encompassed a wide range of healthcare environments including intellectual disability specialist hospital services (n=1), intellectual disability specialist community services (n=3), intellectual disability specialist community and hospital services (n=1), intellectual disability specialist mental health services (n=1), intellectual disability specialist epilepsy clinic (n=1), autism specialist primary care services (n=1). Studies of mainstream/general health services included children’s hospitals (n=2), general hospitals (n=2), general antenatal services (n=1) and an entire health service (n=1). Additionally, two studies, focused on autism and general healthcare.

The age group varied in studies, with six focused on adults and three focused on children. Several studies did not provide precise descriptions of demographics, requiring extrapolation to understand the contextual application of the study.

Across the 16 studies, several service evaluation methodologies were represented, including audits of services (n=10), continuous practice improvement (n=3), questionnaires (n=2) or mixed methods (n=1).

Most studies were multidisciplinary, however two studies (Harrison and Willis, 2015; Marsden and Giles, 2017) focused on nurses’ promotion of inclusive healthcare environments. Three studies were specifically related to paediatrics. Encouragingly, many studies detailed audit and evaluation processes inclusive of methodologies to include perspectives of people with intellectual disability and/or autism (Kennedy et al., 2016; Marsden and Giles, 2017; Pratt et al., 2012; Saqr et al., 2018; Sheehan et al., 2016)

The audit items included in the studies were then analysed to identify the factors used when auditing mainstream healthcare settings. From the many audit items identified within the studies, the factors most relevant to evaluating the healthcare environment were extracted (Table 3). Ten broad factors representing audit items emerged: (A) communication; (B) working together with families/caregivers; (C) supporting decision making; (D) adjusting environments of care; (E) systematic identification to trigger needs assessment; (F) supporting positive behaviour; (G) staff knowledge; (H) having resources for care; (I) management of physical/ medical care; and (J) care management.

The ten factors identified in the analysis of the reviewed studies were then further refined into six conceptual domains, forming a framework for specifically auditing healthcare environments to support the needs of people with intellectual disability and/or autism (Table 4) and unpacked in the evidence for practice, and recommendations for further research sections. The six key domains conceptualised for the audit framework were: (1) understanding key care imperatives, (2) communication to individuals with intellectual disability and/or autism, (3) communication from individuals with intellectual disability and/or autism, (4) providing supportive environments of care, (5) supporting positive behaviour of individuals, and (6) actions to make things go well for healthcare provision.

**Table 4.** Framework for the audit of healthcare environments to support the needs of people with intellectual disability and/or autism.

Audit Domain	Description	A. Communication	B. Working together with families/caregivers	C. Supporting decision making	D. Adjusting environments of care	E. Systematic identification to trigger needs assessment	F. Supporting positive behaviour	G. Staff knowledge	H. Having resources for care	I. Management of physical/ medical care	J. Care management
(1) Understanding key care imperatives	• Philosophy of care: valuing the person as an individual • Understanding the role of government reform & policy in promoting the rights of people with disability • Legal & ethical frameworks for healthcare delivery • Relationship-centered care & collaboration		✓		✓		✓	✓			
(2) Communication to individuals with ID and/or autism	• Awareness of the balance between expressive & receptive language • Understand the need to accurately assess- communication ability & preferred communication platform • Provide information in accessible format & straightforward language • Involve caregivers in communication interactions • Adjust communication to provide concrete instructions in sequential order • Respecting the individual’s right to communicate in the first instance, prior to deferring to caregivers • Awareness that poor communication leads to increased risk of adverse outcomes & poor uptake of healthcare services	✓	✓					✓			
(3) Communication from individuals with ID and/or autism	• Awareness of expressive language skills (A); (B); (G) • Allowing more time to listen actively to individuals with ID &/or autism • Awareness of acquiescence • Understanding non-verbal language • Collaborating with caregivers to understand communication from individuals with ID &/or autism • Recognizing that behaviour is a form of communication	✓	✓					✓			
(4) Providing supportive environments of care	• Make reasonable adjustments to environments • Identify the need & type of wayfinding in the environment that can be facilitated • Understand that individuals with ID &/or autism prefer to routine & need to be consciously taught new routines • Adjust the environment to reduce the impact of anxiety on behaviour of individuals with ID &/or autism				✓				✓		
(5) Supporting positive behaviour of individuals	• Understand how to teach functional routines & behaviours appropriate to the healthcare environment the unwritten social rules of the environment • Understanding overstimulation from the environment & sensory triggers for individuals with autism • Understand the role of anxiety in supporting positive behaviour • Focusing on preventative measures to avoid challenging behaviours in susceptible individuals with ID &/or autism • Reinforcing positive behaviour • Minimizing restrictive practices						✓	✓			
(6) Actions to make things go well for healthcare provision.	• Asking about individualized care needs • Allowing extra time for care • Making formal plans for reasonable adjustments • Organizing resources in advance for reasonable adjustments • Planning in advance to reduce stressful situations • Making spontaneous reasonable adjustments • Planning multidisciplinary input for reasonable adjustments • Minimizing anxiety due to waiting for care	✓	✓		✓	✓			✓		✓

### Advances in knowledge

Importantly, positive healthcare outcomes and identification of service gaps resulted from the audit process in several included studies, supporting the notion that auditing healthcare environments is an important process for increasing equitable healthcare access (Foy et al., 2020). Positive healthcare outcomes included improved prescribing and provision of psychological interventions for people with intellectual disability and depression (da Costa et al., 2011), better identification of individual needs and provision of supports (Kennedy et al., 2016), greater overall adherence to best practice for adults with intellectual disability and epilepsy (Esan and Marker, 2010) and improvements to positive behaviour support planning, dysphasia planning, and patient environments (Penney and Blair, 2020). Auditing also found service gaps, including limitations to health professionals’ knowledge and confidence (Pratt et al., 2012); environmental barriers in waiting rooms (Saqr et al., 2018); specialised health needs not being noted, and difficulties with multidisciplinary liaison (Cocquyt, 2018).

### Gaps identified in knowledge

Although the improvements resulting from audit processes are promising, this review has also identified that a major limitation across the studies reviewed is the poor reporting of methodological procedures or measured outcomes as part of the study design, affecting quality of the evidence. Additionally, this lack of methodological reporting impacts the findings of this scoping review. Further, as most of the studies were from the UK and/or based in disability-specific services, insights into how the multiple dimensions of healthcare environments impact people with intellectual disability and/or autism in other contexts has received little attention. This concentration of studies in specialised disability-specific services may impact the quality of findings as a myriad of environmental factors within the range of mainstream healthcare contexts could affect the experience of people with intellectual disability and/or autism.

The analysis of included studies indicates that most auditing processes focused on body structures or systems rather than the interactions of person and environment factors. Most studies comprised primarily of checklists or audits of the healthcare actions or medical care provided in healthcare environments. Only one study detailed an environmental audit designed specifically to improve the experience of people with autism attending UK hospital and community healthcare facilities, focusing on sensory and communication issues (Simpson, 2015). No studies were identified that could assist health providers to holistically audit all the environmental elements categorised by the ICF that could be present in healthcare environments and impact health outcomes for people with intellectual disability and/or autism. There is a disparate balance of studies globally, and as Australian researchers, the absence of any Australian studies is of major concern. Although Australia is a signatory to the global conventions that promote equity and inclusion and the removal of barriers to healthcare, it is unclear whether the focus is on physical environments, rather than barriers arising from the less tangible elements of the environment, such as support and relationships, attitudes and services, systems and policies detailed in the ICF (WHO, 2001), and unpacked by Schneidert et al. (2003).

### Evidence for practice and research recommendations

To our knowledge, this scoping review is the first to explore how healthcare environments can be evaluated using audit tools to provide improved healthcare access and outcomes for people with intellectual disability and/or autism. The evidence and audit items identified in this scoping review of the literature, forms the basis for the proposed conceptual framework of six domains for an audit tool to evaluate healthcare environments, unpacked below, that seeks to promote better health outcomes for people with intellectual disability and/or autism.

#### Understanding key care imperatives

This first domain focuses on the need to provide a philosophy of care oriented around valuing and respecting the person as an individual at the centre of a network of collaborative relationships for care. This domain encompasses two ICF environmental factors: attitudes; and services, systems and policies of healthcare environments. The legal and ethical frameworks guiding healthcare practice should be framed around recognising the rights of people with a disability. These fundamental care imperatives, particularly in relation to collaborating with caregivers, decision making and consent practices, were central in most studies reviewed (see Table 3). Examples of audit items in reviewed studies that enabled a positive experience of healthcare included consultation and involvement in treatment, respecting decisions, consideration of cultural and social background (Chilvers et al., 2013), considering the experience of the person and the family (Davies et al., 2019), supporting choices and decision making (Marsden and Giles, 2017; Sheehan et al., 2016) and addressing staff attitudes (Raymaker et al., 2017).

#### Communication to individuals with intellectual disability and/or autism

This domain focuses on the need for health professionals to accurately establish the communication preferences of the person, adopt them in all interactions and collaborate with caregivers. This also involves promoting the provision of healthcare information in accessible and straightforward language. This domain relates to two ICF environmental factors: support and relationships; and services, systems and policies. People with intellectual disability can have difficulty with speed and amount of information delivered, or being talked over by health professionals (Ali et al., 2013). Similarly, people with autism can have slower thinking and information processing when interacting with professionals or may prefer non-verbal communication (Mason et al., 2019). Collaborating with caregivers is important to clarify communication strategies which actively involve the person with intellectual disability and/or autism in their healthcare (Lewis et al., 2017; Simpson, 2020). Specific issues around communication were not separated into understanding communication from the person versus health professionals communicating in an accessible way, in the reviewed studies, which instead focused on staff awareness or understanding, and communication in general. Examples of audit items identified in the studies which support this domain include providing accessible patient education and explaining medication (da Costa et al., 2011; Esan and Marker, 2010), allowing for communication needs (Simpson, 2015; Simpson, 2020), and having family support during healthcare services (Chilvers et al., 2013). Adjustment of communications made to individuals with intellectual disability and/or autism was an important evaluation of healthcare quality in most of the studies reviewed.

#### Communication from individuals with intellectual disability and/or autism

This domain requires health professional’s recognition of the role of expressive language skills, nonverbal language, and behaviour in communication (Foley and Trollor, 2015) and encompasses two ICF environmental factors: support and relationships; and services, systems and policies. Examples of audit items in the reviewed studies included undertaking communication assessment (Cocquyt, 2018), identifying communication needs (Pratt et al., 2012; Saqr et al., 2018), and identifying communication and care needs using a health passport (Penney and Blair, 2020; Sheehan et al., 2016). This domain was seen as separate to health professional understanding of how to communicate *to* people with intellectual disability and/or autism because key issues include understanding the potential for acquiescence (Beail, 2002) and understanding nonverbal communication and behaviour in the context of diagnostic overshadowing (Jamieson and Mason, 2019). Acquiescence refers to the risk that people with intellectual disability and/or autism may respond “yes” to a question without necessarily understanding its meaning (Beail, 2002). Diagnostic overshadowing refers to the symptoms of an underlying health condition being attributed to intellectual disability and/or autism. The risk is that health professionals may misinterpret nonverbal communication, or unusual behaviour that people with intellectual disability and/or autism use to communicate pain or other symptoms (Jamieson and Mason, 2019). Environmental changes for avoiding acquiescence and diagnostic overshadowing include adapting health professionals’ communication styles, using standardized questionnaires that are appropriate for non-typical thinkers and/or communicators (Stancliffe et al., 2014) and collaborating with caregivers to better understand communication from individuals with intellectual disability and/or autism (Foley and Trollor, 2015; Ziviani et al., 2004). These specific issues impacting how health professionals understand verbal and non-verbal communication from people with intellectual disability and/or autism were not specifically evaluated in the reviewed studies, but reflected audit items focused on focused on staff awareness or understanding, and communication in general.

#### Providing supportive environments of care

The fourth domain focuses on making reasonable adjustments to healthcare settings. This includes identifying requirements for wayfinding in the environment, adjusting routines in the healthcare environment, adjusting physical design of healthcare spaces, adjusting sensory stimuli, and adjusting the environmental demand for social interaction or social stimuli that can increase anxiety and associated behavioural responses. This domain encompasses several ICF environmental factors: products and technology; human environments; and services, systems, and policies. Examples of audit items in the selected studies included considering the accessibility of clinic spaces, providing alternatives to overwhelming waiting room environments (Raymaker et al., 2017), reorganising sensory aspects of the environment (Penney and Blair, 2020), asking about sensory sensitivities and providing an environment where stressful situations can be escaped, such as a quieter and more private area (Simpson, 2020). A focus on evaluating provision of reasonable adjustments to the environments of healthcare was identified in several of the reviewed studies, especially in relation to sensory and psychosocial issues. Bright lights and loud noises can be sensory triggers, causing discomfort for individuals with autism (Saqr et al., 2018; Simpson, 2020) and can be exacerbated by visual overload from the clutter of posters on walls (Simpson, 2020; Walsh et al., 2020). Additionally, sitting in waiting rooms for extended periods is reported by people with autism to be challenging (Saqr et al., 2018; Simpson, 2020), and further aggravated by the proximity of other people (Mason et al., 2019) or delays to care (Walsh et al., 2020). Behaviours of people with autism arising from over-stimulation in waiting rooms can be misinterpreted by healthcare workers as attention-seeking or a tantrum (Raymaker et al., 2017).

#### Supporting positive behaviour of individuals

This domain involves healthcare practices which recognise the need to support individuals with intellectual disability and/or autism, aimed at reducing negative responses and promoting positive behaviour. It focuses on practices which recognise the relationship between anxiety and behaviour and provide reasonable adjustments to accommodate individual sensory and social needs. This domain relates to several ICF environmental factors: support and relationships; attitudes; and services, systems, and policies. Examples of audit items in the reviewed studies includes assessing individual needs to provide effective interventions and use least restrictive practice (Davies et al., 2019); assessing behavioural needs and utilising special interests to assist in care (Pratt et al., 2012); ensuring a positive support plan is in place (Penney and Blair, 2020); and meeting sensory needs (Raymaker et al., 2017). Healthcare settings and routines of healthcare can increase anxiety for people with intellectual disability and/or autism and sometimes behaviour challenges can be an expression of underlying distress. Supporting positive behaviour addresses these challenges by identifying the challenging behaviour, analysing the underlying stressor/anxiety that may be contributing to the behaviour, formulating, and implementing strategies to support positive behaviour, including parents/caregivers in decision-making, and monitoring change (Hieneman, 2015).

#### Actions to make things go well for healthcare provision

The sixth and final domain focuses on overall practices which promote positive experiences of healthcare; practices which are individualised, enact reasonable adjustments, and are resourced for the additional time and resources required for caring for individuals with intellectual disability and/or autism. This domain relates to the ICF factor of services, systems, and policies. Planning the delivery of care in the complex healthcare environment was frequently identified in reviewed studies, categorised under the factor of care management. Audit items identified in the reviewed studies included arranging access to services that best meet the needs of children and including home/community liaison (Chilvers et al., 2013); communication with primary care physician and review by multidisciplinary team (Cocquyt, 2018); managing and coordinating care during admission (Davies et al., 2019; Marsden and Giles, 2017); identification of diagnosis at intake (Harrison and Willis, 2015; Kennedy et al., 2016; Pratt et al., 2012); preparation for admission (Kennedy et al., 2016); ensuring a person centred plan is in place (Penney and Blair, 2020); and having a key professional or nurse liaison role to plan overall care or discharge (Kennedy et al., 2016; Shardlow and Thalayasingam, 2010; Sheehan et al., 2016).

#### Methodological and research considerations

There are a few limitations of this review: exclusion of articles not written in English, and the inability to report detailed demographics to make meaningful comparisons due to inadequate reporting in some of the included studies. Several studies (Cocquyt, 2018; Esan and Marker, 2010; Kennedy et al., 2016; Sheehan et al., 2016; Simpson, 2015; Simpson, 2020; Penney and Blair, 2020) reviewed did not clearly describe the processes of audit tool development, affecting rigour. An important further consideration for audit development is intellectual disability, autism and dual diagnosis of both are not a universal experience, and individuals present with a different mix of sensory, communication, physical, social, behavioural and family support factors. These result in variable healthcare barriers, which requires additional sensitivity of audit tools. For instance, the needs experienced by someone with profound intellectual disability and multiple chronic and complex health problems who requires 24-hour care will vary compared to someone with borderline intellectual disability who lives independently with only drop-in support (Wilson et al., 2020). Additionally, the barriers to healthcare may be based on the differing thinking and information processing styles that constitute each of the forms of neurodiversity. While ASD represents fundamental differences in thinking as compared to typical thinking ID represents difference in the volume of information that can be managed at any time and the speed at which processing occurs (Cashin, 2020). Despite the pathway to the barriers differing, the adjustments required are in many cases very similar. Future research should ensure maximum demographic variables are captured, as well as the context of health settings and participants’ lives, to enable greater comparison and/or replication. The altering landscape of service provision and use of technology, including the increased utilisation of telehealth during the COVID-19 pandemic, and the enduring impact this has on healthcare accessibility for people with intellectual disability and autism should be a consideration in future audit tools (Friedman, 2022; Rawlings et al., 2021).

## Conclusion

Our review of the literature indicated that there appeared to be no comprehensive audit tools to assist health services to provide a supportive environment for people with intellectual disability and/or autism. This scoping review is the first to describe how healthcare is audited for people with intellectual disability and/or autism. The scoping review identified that current audits of healthcare contexts address either intellectual disability or autism with insufficient reporting of methods used to develop the audit and outcome measures. These evaluations are frequently completed in specific specialty healthcare contexts, focusing on biomedical aspects of body functions and structures. Additionally, greater engagement with international frameworks, such as the ICF framework that incorporates the individual and the environmental contexts, could improve the design and study of future audit tools. The framework of six domains that emerged from the scoping review data offers a starting point to further develop an audit tool that is inclusive of both intellectual disability and autism. Further research is needed to develop audit tools that capture the broad environmental factors impacting equity and access to healthcare for people with intellectual disability and/or autism.

## Supplemental Material

Supplemental Material - A scoping review to inform an auditing framework evaluating healthcare environments for inclusion of people with intellectual disability and/or autism Supplemental Material for A scoping review to inform an auditing framework evaluating healthcare environments for inclusion of people with intellectual disability and/or autism by Michelle Kersten, Nathan John Wilson, PhD, Amy Pracilio, Virginia Howie, Julianne Trollor, Thomas Buckley, Julia Morphet, Julianne Bryce, Ken Griffin and Andrew Cashin, PhD in Journal of Intellectual Disabilities
