# Supplementary material for: A scoping review to inform an auditing framework evaluating healthcare environments for inclusion of people with intellectual disability and/or autism
Source: J Intellect Disabil. 2023 May 22;30(1):173–95. doi: 10.1177/17446295231174282 (PMC13049222; doi:10.1177/17446295231174282)
Supplement: Supplemental Material - A scoping review to inform an auditing framework evaluating healthcare environments for inclusion of people with intellectual disability and/or autism [file sj-pdf-1-jld-10.1177_17446295231174282.pdf]

Supplementary Table

Overview of the included papers

Author, year & country	Study Aim	Number of participants	Context or setting	Methodology	Relevant Findings
Chilvers et al. (2013) UK	Assessed specialist national mental health services, measured against model service standards from Royal College of Psychiatry	Parents n=26<br>Children n= 8, mean age= 12.8 male = 69.2%	Highly specialised national children & adolescent outpatients service for developmental delay & mental health diagnoses.	Parent & child questionnaires based on Royal College of Psychiatrists model of service, assessed whether service met user needs.	Positive service performance measured by consultation & involvement in treatment; consideration of cultural & social background, access to a service best meeting needs of child; inclusion of home/school reports or visits, family support & liaison with other services.
Cocquyt (2018) UK	Audit of quality and development of multidisciplinary care pathway for health care for people with profound & multiple learning disability	Multiple audits completed:<br>2014 n=35<br>2015 n=not reported<br>2016 n=not reported<br>2017 n=not reported	Regional community adult ID service	Audits based on a range of UK recommendations for healthcare for people with ID.<br>Audit of electronic patient record.	**Outcome:** Specialist health needs not identified & multidisciplinary interventions lack team liaison.
da Costa et al. (2011) UK	Audit of management of depression in community services to NICE guidelines	Multiple audits completed:<br>Audit 1 n=34<br>Audit 2 n=39	Specialist ID disability community services, age not reported, but appears to be adults.	Audit based on NICE guidelines for management of depression.	Audit tool developed including short term psychological treatment & medication.<br>**Outcome**: Improved prescribing & provision of psychological interventions.
Davies et al. (2019) UK	Audit specialist inpatient mental health unit adherence to positive behaviour support standards.	n=2 staff groups	Specialist mental health unit for people with ID. Age not reported, but appears to be adults.	Audit of a single service based on the Positive Behavioural Support Academy standards.	Audit tool developed assessing domains of positive behaviour support. Audit question development & final audit tool not reported.<br>**Outcome**: Recommended measuring service via a Likert scale to capture progress.
Esan and Marker (2010)	Audit of specialist epilepsy clinic to	n=45<br>Male 47%	Specialist multidisciplinary	Audit & follow-up audit of service adherence to NICE	Audit tool developed by the service based on NICE standards.

Author, year & country	Study Aim	Number of participants	Context or setting	Methodology	Relevant Findings
UK	NICE guidelines.	Female 53%<br>Mild ID 38%<br>Moderate ID 13%<br>Severe ID 20%<br>Not specified 29%	epilepsy clinic for people with ID.<br>Outpatients department of district hospital.<br>Age not reported, but appears to be adults.	guidelines, via retrospective data extraction from GP letter in the medical record.	**Outcome**: Improvements in service adherence overall, however local service & resource constraints were identified.
Harrison and Willis (2015)<br>UK	Audit of antenatal services to identify service provision patterns for pregnant women with ID.	n=10 general practices, with low response rate of 6.3%<br>n=2 hospital trusts	Antenatal services.<br>ID, for age not reported, but appears to be adults	Audit carried out by primary care liaison nurses, & learning disability nurses based at local hospitals..	**Audit outcome**: Mothers or fathers with ID are not systematically identified, lack of practice guidelines, with potential impacts on pregnancy & parenting outcomes for people with ID.
Kennedy et al. (2016)<br>UK	Five-year continuous quality improvement process to raise awareness and redesign hosptial care provision	Patient n not reported.	Children's hospital.<br>Autism, age not reported	Case study approach utilizing stakeholder conference, audit, questionnaires & feedback from families, staff & autism champion staff, to produce patient care improvements.	An audit tool is referred to but not specifically reported on.<br>**Evaluation outcome**: identification of autism at preadmission, advance preparation, information sharing & collaboration with parents, school & community services are key to providing safe, individualised care; education staff better prepared for communication & understanding behaviour; toolkit developed for autism education strategy, autism champion role, & autism care standards.
Marsden and Giles (2017)<br>UK	Identification of nursing challenges to produce a framework for making reasonable adjustments to nursing	n=3 hospitals	Acute general hospitals.<br>ID, age not reported, but appears to be adults	Guba & Lincoln evaluation approach, to change culture & increase learning including 3 senior staff meetings, 3 further staff meetings to thematically analyze data, & further meetings with staff & people with ID to create a reasonable adjustments framework.	Formal audit tool not undertaken<br>**Outcome**: Four themes for reasonable adjustments identified

Author, year & country	Study Aim	Number of participants	Context or setting	Methodology	Relevant Findings
Penney and Blair (2020) UK	Adaptation of a care quality audit tool for use in learning disability services.	n=4 in audit team working group (including quality leader, learning disability nurse, and 2 volunteers).	Learning disability services	Adaptation of original SCAPE audit tool (safe, clean and personal every time) which was initially a nursing tool utilised in acute nursing settings, later adatped for broader social care use. Use of a working group to oversee development, pilot and implementation of new tool.	Three new standards added to original SCAPE tool appropriate for learning disabilities: epilespy care & treatment; hospital care for people with learning disabilities; healthcare for people with learning disablities. Additionally, "red" questions for acute version of the tool adapted to include more learning disability appropriate items: use of a person centred plan; attendance at an annual health check; dysphasia risk management & when relevant advice from a language/speech therapist; use of behaviour support plan, and monthly check to ensure plan adherence; staff understand health passport use & use. **Outcome:** Tool piloted in 2018, & implemented in 2019 with good results. Initial low uptake of behaviour support plans in one area, later rectified at 100% uptake. Initial concerns with environment, improved within four months at another site.
Pratt et al. (2012) UK	To develop an intervention to improve healthcare, measured using a baseline & post intervention audit.	Baseline audit: n not reported. Post intervention audit: n=20 staff n=8 families interviewed	Children's hospital, including emergency, intensive care & planned surgery. autism &/ or ID or children with challenging behaviours.	Continuous quality improvement methodology, including initial audit, focus group of families & expert panel to develop principles for modifying admission processes.	Preadmission checklist and written admission plan communicated to ward staff, play specialist, anesthetics & medical team. **Outcome**: Initial audit identified nurses have anxiety managing challenging behaviours & feel a lack of knowledge about autism; caregivers identify problems during admission could be avoided with adjusting planned admissions. Post audit outcomes were identified as positive, but no specific data presented.
Raymaker et al. (2017) US	To develop a barriers to healthcare standardized tool	Self-administered internet questionnaire: n=209 autism n=55 disability & no autism n=173 controls	Internet survey of adult members of the AASPIRE autism network. Mean age 37	An existing healthcare barrier checklist was modified for autism, tested on the sample & modified.	Participatory methodology to identify & compare barriers to healthcare for autism and non autism controls. **Outcome**: Barriers to healthcare: anxiety/ fear (35%), difficulties with information processing during discussions with healthcare providers (32%), healthcare costs (30%), sensory issues at

Author, year & country	Study Aim	Number of participants	Context or setting	Methodology	Relevant Findings
					healthcare appointment (30%), & difficulty communicating with healthcare provider (29%).
Saqr et al. (2018) US	To understand health disparities in access & delivery of primary care	Focus groups of adults/ adolescents n=10 (9 male, 1 female) Retrospective chart review n=74	Primary care for adults & adolescents on autism. Mean age 21.2	(1) Retrospective chart review, including pre-visit questionnaire regarding reasonable adjustment needs; (2) focus group.	Frequent barriers requiring reasonable adjustment: difficulty with waiting or the waiting room (n=12), dislike of loud noise or children crying (n=11), aversion to needles (n=6), dislike of touch (n=6). Focus group: sensory sensitivity, anxiety from waiting for care, lack of mutual understanding, communication & trust. **Outcome**: waiting & waiting room is a key barrier. A negative feedback loop of fear prior to coming to clinic was identified, linked to anticipation of social interaction, adding to heightened sensory sensitivity, & making it harder to communicate & interact socially.
Shardlow and Thalayasingam (2010) UK	Audit on physical health care monitoring in tertiary service based on Royal College of Psychiatry standards	n=33 service users with ID, significant behaviour impairment, autism, mental illness & physical disorders.	Specialist residential service for long stay inpatients with ID & challenging behaviours, for age not reported, but appears to be adults.	Retrospective file audit & staff discussion.	Developed audit tool to examine adherence to practice standards for physical healthcare monitoring of service users using standards from the Royal College of Psychiatrists & local hospital trust policies.
Sheehan et al. (2016) UK	Feasibility study for a future national audit of inpatient care for people with ID.	n=9 acute general hospital trusts n=6 mental health services n=176 retrospective file audits 56% male, 48% female Mild- moderate ID 45%; Severe-profound ID 21%; Unknown ID level 34%	People with ID accessing acute general hospital or mental health services. Mean age=43	Audit tool & criteria developed with advisory group containing people with ID, caregivers & professionals; & informed by literature.	**Outcome:** Poor documentation of dysphagia/ epilepsy assessments, caregiver needs for discharge planning; & positive outcomes when families are involved in discharge planning, most standards were met.

Author, year & country	Study Aim	Number of participants	Context or setting	Methodology	Relevant Findings
Simpson, 2020) UK	Study to (a) develop audit tool & (b) pilot audit of physical healthcare environments to improve accessibility	n=not reported	People with autism accessing healthcare locations, for age not reported, but appears to be for adult & children.	Methodology for developing tool not described. Tool developed by an NHS trust for nurses & healthcare professionals.	**Outcome:** Most environmental changes are inexpensive & simple; however, it is difficult to meet all individual needs. Positive changes identified included awareness training for reception & front-line staff, providing dimmer lighting, eliminating smells, replacing loud ticking clocks, better signage, a comfortable temperature, providing quiet waiting areas, assessing communication needs, & arranging individual reasonable adjustments.
Tromans et al. (2019) UK	To determine compliance to end of life care standards for people with ID & dementia.	n=32 records of service users with ID & comorbid dementia	ID service including community multidisciplinary team & inpatient mental health unit. Mean age=58.7	Retrospective file audit: Audit criteria were developed referencing clinical guidelines, resulting in 24 medical criteria.	**Outcome**: Overall lack of involvement of palliative care team for people requiring end of life care for ID & comorbid dementia.
